# Salvage Fixation With a Single Nitinol Compression Staple for a Lateralized Fibular Tunnel in Posterolateral Corner Reconstruction of the Knee

**DOI:** 10.1016/j.eats.2021.12.023

**Published:** 2022-03-28

**Authors:** Nathan Fackler, Theofilos Karasavvidis, Arya Amirhekmat, Dean Wang

**Affiliations:** a University of California Irvine Department of Orthopaedic Surgery, Orange, California, U.S.A.; b Georgetown University School of Medicine, Washington, D.C., U.S.A.

## Abstract

Recognition and treatment of injuries to the posterolateral (PLC) corner of the knee have increased in recent decades. Despite advancements in surgical technique, complications can occur in up to 20% of PLC reconstructions. Lateralization of the fibular tunnel during drilling is a common intraoperative pitfall and can lead to cortical breach or fibular head fracture after graft tensioning. Compression staples have been increasingly used for fracture fixation in the hand, foot, and ankle. Because of its pseudo-elastic properties, insertion of a low-profile, compressive Nitinol staple could serve as an intraoperative salvage technique to reinforce and prevent failure through a thin lateral cortex of the fibular head. The purpose of this Technical Note is to describe a reproducible technique for the implementation of a Nitinol staple for reinforcement of a lateralized fibular tunnel in posterolateral corner reconstruction.

## Introduction

The posterolateral corner (PLC) of the knee consists of stabilizing structures that resist varus and external tibial rotation forces through all ranges of motion.^1^ The major stabilizers of the PLC are the fibular collateral ligament (FCL), popliteofibular ligament (PFL), and popliteus tendon.^2^ Injuries of the PLC account for ∼16% of all ligamentous injuries and often occur in combination with other ligamentous knee injuries as a result of high-energy trauma involving hyperextension, twisting of the knee or direct varus stress, causing rotational and varus instability.^3^

Several surgical techniques for addressing PLC injuries have been described, including repair and reconstruction of the PLC. As compared to repair, reconstruction is considered the superior technique and has been associated with lower reoperation rates.^4^ In PLC reconstruction, the fibular tunnel is created in an anterolateral-to-posteromedial fashion in the fibular head in order to maximize socket length and construct stability. Early methods of reconstruction have aimed to drill the fibular tunnel through the centers of the anatomic footprints of the FCL and PFL. However, more recent studies have shown that this can lead to a shallow tunnel that is vulnerable to lateral blowout. To avoid this, a modified technique was proposed that involved starting drilling at the margins of the footprints to maximize the amount of lateral cortex and increase the mechanical stability of the fibular tunnel.^5^ Despite this adjustment in technique, misplacement of the tunnel, either too lateral or too superior, remains a common intraoperative pitfall, resulting in a thinned lateral fibular cortex and possible subsequent cortical breach and graft failure.^6^

Nitinol staples have gained popularity because of their unique pseudoelastic properties and ability to dynamically compress materials, combined with ease and speed of insertion.^7^ They are frequently used as a compressive fixation method in foot, ankle, and hand surgery and their applications are rapidly expanding in orthopaedic surgery.^7^ Intraoperative salvage techniques in the setting of a thinned lateral cortex or tunnel breach have rarely been described.^8^ The following Technical Note aims to describe a salvage fixation method with a single Nitinol staple for a lateralized fibular tunnel in posterolateral corner reconstruction of the knee.

## Surgical Approach to Fibular Head (Fig 1)

The surgical approach to the fibular head for PLC reconstruction has been well described.^8^ In short, a lateral incision is made along the iliotibial band and extended distally to the space between the fibular head and Gerdy tubercle. Careful neurolysis of the common peroneal nerve is then performed. Posterolateral dissection is performed next via blunt dissection of the lateral gastrocnemius tendon and soleus to expose the posteromedial aspect of the fibular styloid and the popliteus myotendinous junction. Subperiosteal dissection is performed from the anterior to posterior aspect of the lateral fibular head and distally to the level of the champagne glass drop-off of the fibular head. Finally, posterior dissection of the soleus muscle from the posteromedial aspect of the fibular head is performed to expose the location of the posterior aspect of the fibular tunnel.

**Fig 1:**
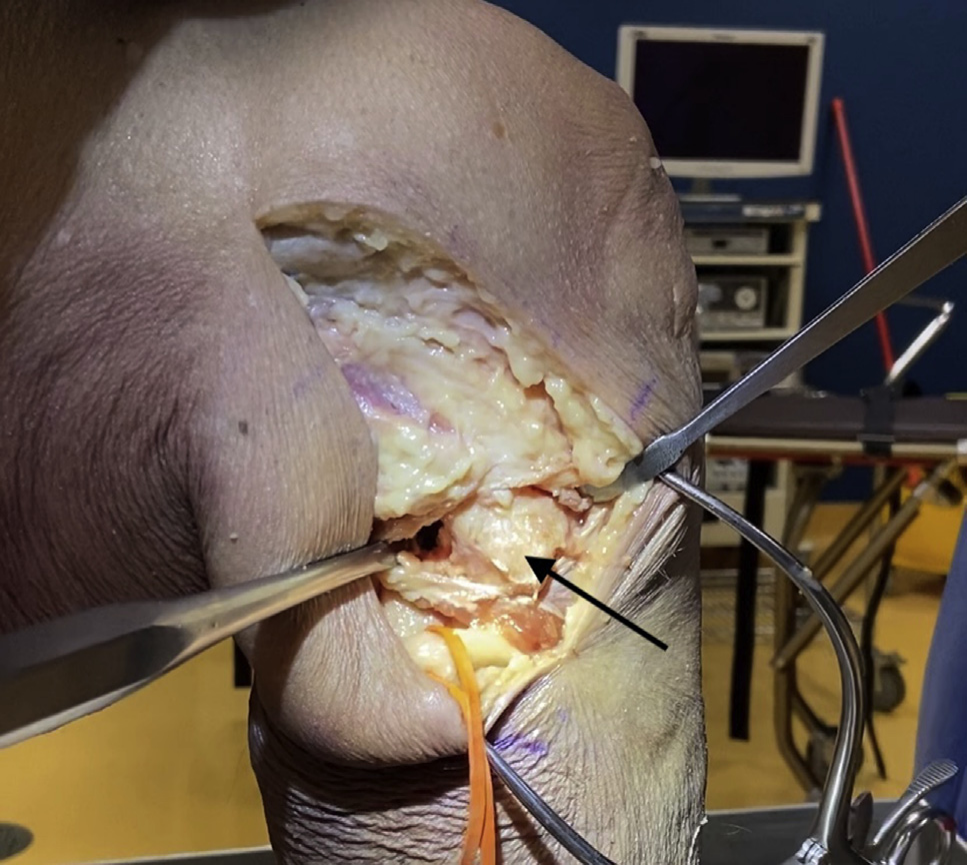
Lateral intraoperative view of a right cadaveric knee demonstrating dissection technique to expose the fibular head (arrow) prior to tunnel drilling in posterolateral corner reconstruction. A surgical vessel loop is used to protect the peroneal nerve.

### Drilling of a Proper Fibular Tunnel (Video 1)

**Fig 2:**
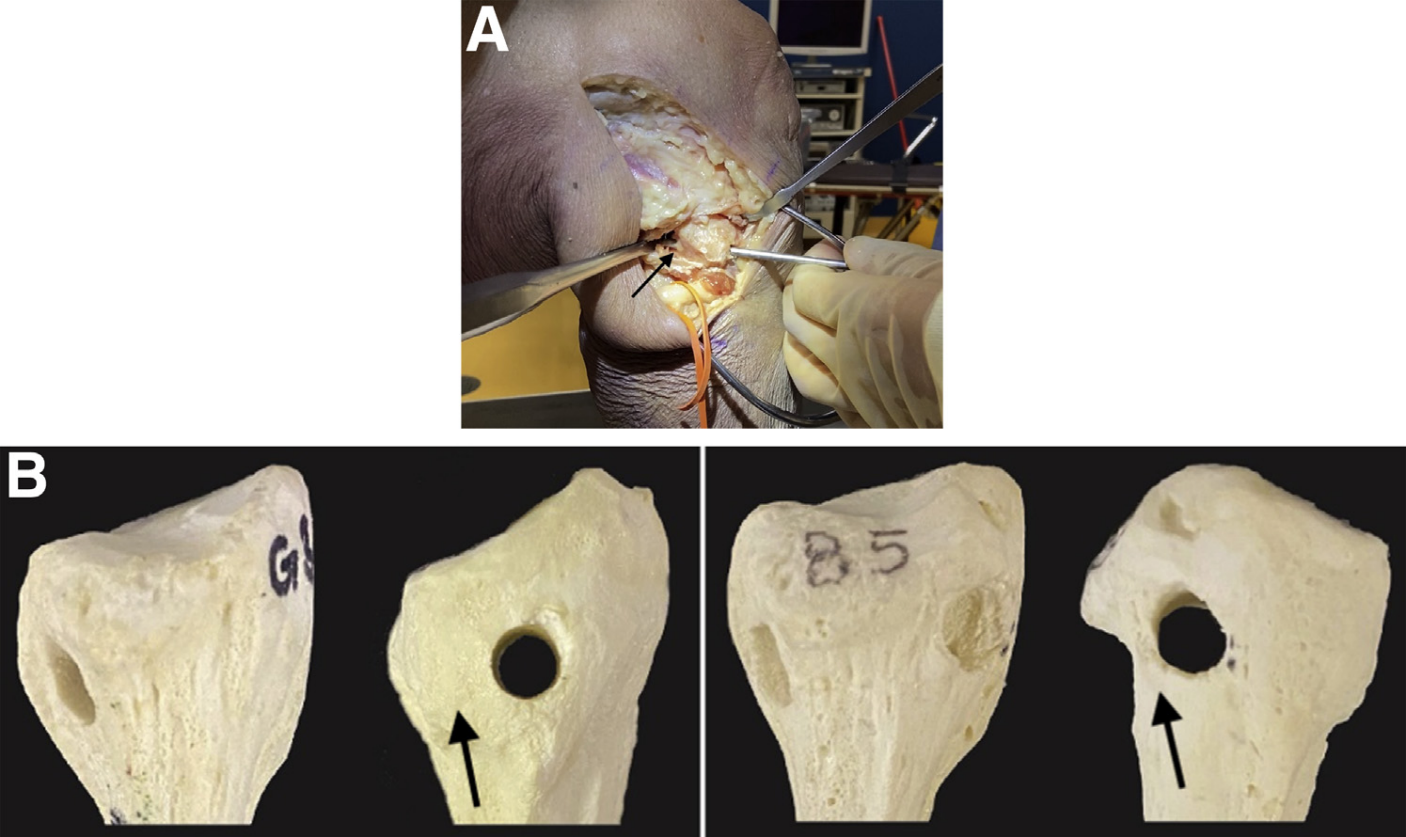
(A) Lateral intraoperative view of a right leg demonstrating improper lateralized drill trajectory (anterolateral-to-posterolateral [arrow]) of the fibular tunnel for posterolateral corner reconstruction. A surgical loop has been placed to identify the peroneal nerve. (B) Sawbone model of a fibula in anterior-posterior and lateral views demonstrating proper (right) and lateralized (left) tunnel drilling for posterolateral corner reconstruction. Arrows have been placed to highlight the difference in width of the lateral cortices.

A guide wire is placed from the anteroinferior margin of the FCL footprint on the anterolateral aspect of the fibular head directly proximal to the champagne drop-off and is drilled posteromedially to the posteroinferior margin of the PFL footprint on the downslope of the fibular styloid. A 7-mm reamer is then used to drill the fibular tunnel bicortically, and a passing suture is placed through the tunnel to facilitate passage of the graft (Fig 2). The graft is then secured with the stitch and passed through the fibular tunnel in a posterior-to-anterior fashion.

### Recognition of Lateralized Fibular Tunnel and Application of Low-Profile Compression Staple

**Fig 3:**
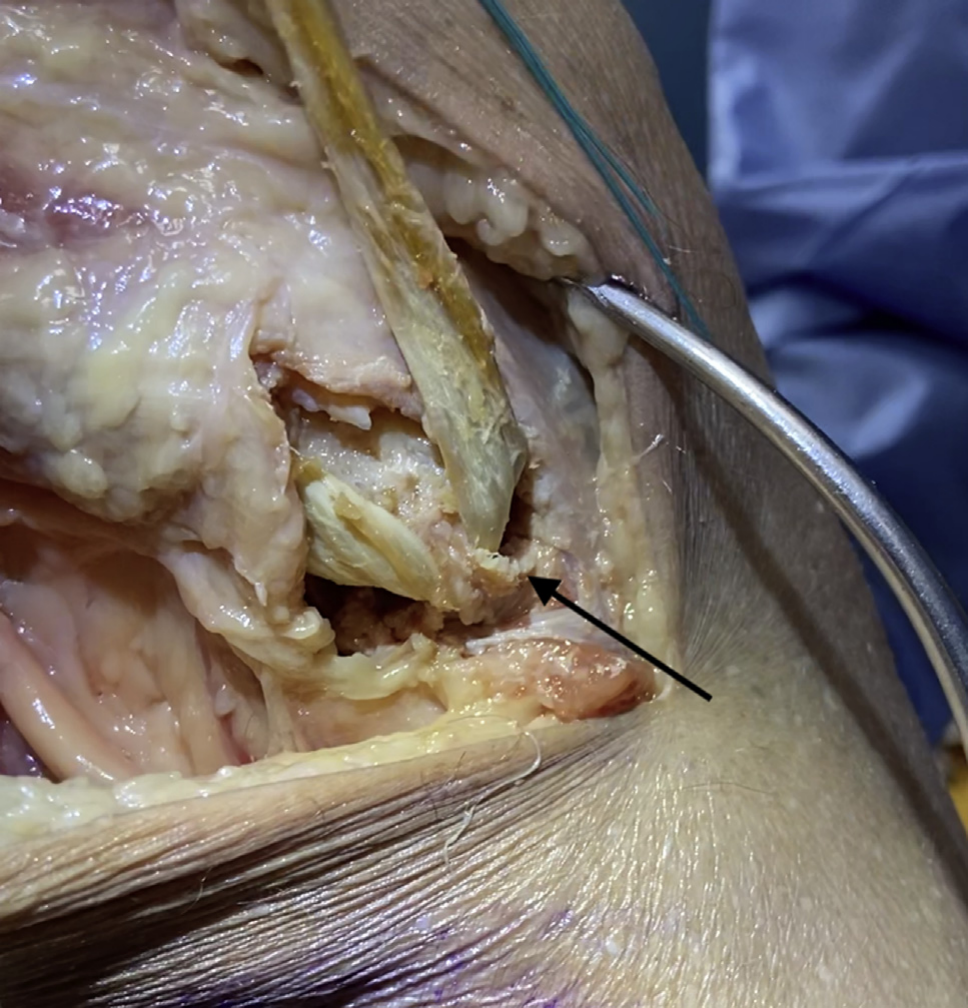
Demonstration of a lateralized fibular tunnel in posterolateral corner reconstruction in the right knee. Graft has been passed through the fibular tunnel and tensioned, leading to a fibular head blowout fracture through the thinned lateral cortex (black arrow).

When the guide wire is mistakenly placed too laterally on the fibular head, lateralization of the fibular head tunnel can occur, resulting in a short tunnel and thin lateral fibular head cortex (Fig 3). Lateral breach of the femoral tunnel can subsequently occur during drilling or graft tensioning (Fig 3). Reinforcement of the lateralized fibular tunnel with a Nitinol staple can help salvage this mispositioned tunnel.

**Fig 4:**
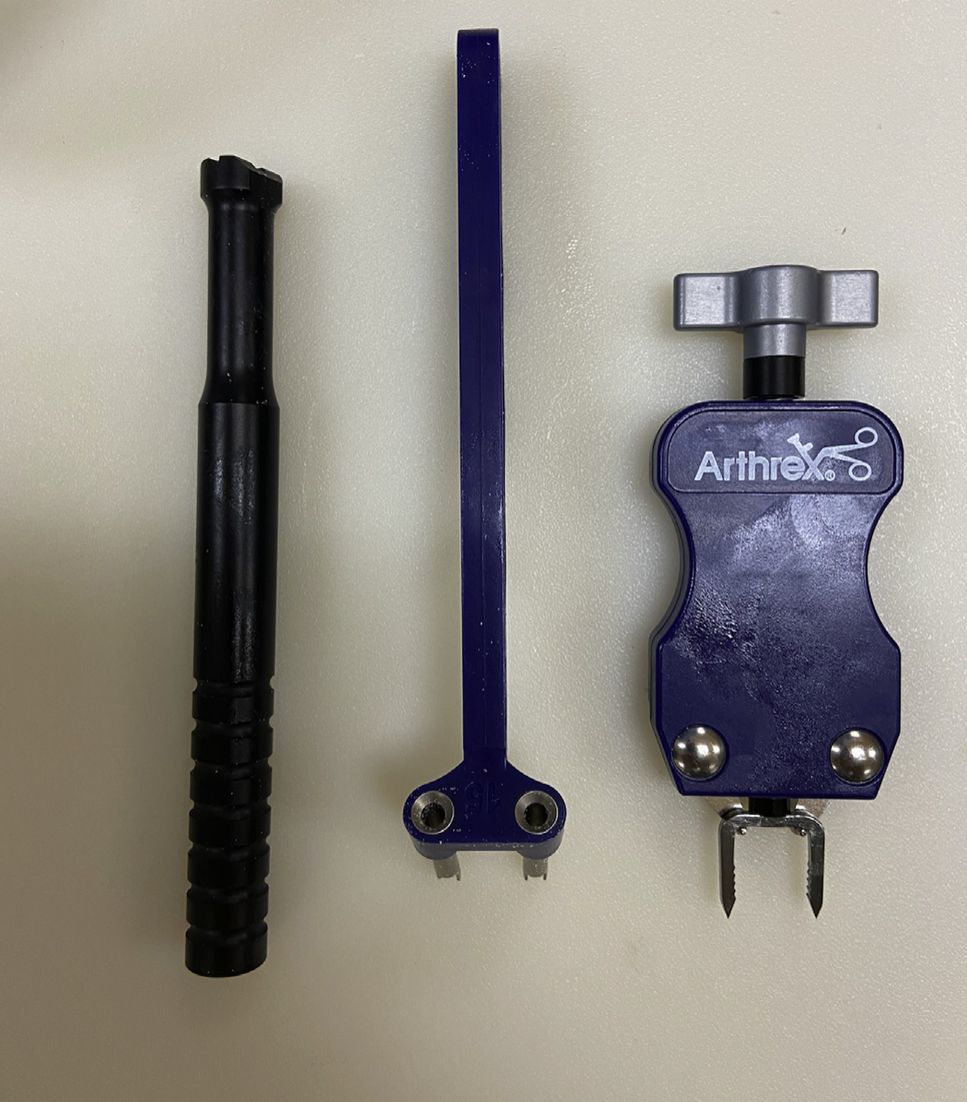
Materials for implementation of the Nitinol staple (Arthrex). Far left, staple tamp; middle, staple drill guide; far right, staple implementation device with nitinol staple attached.

**Fig 5:**
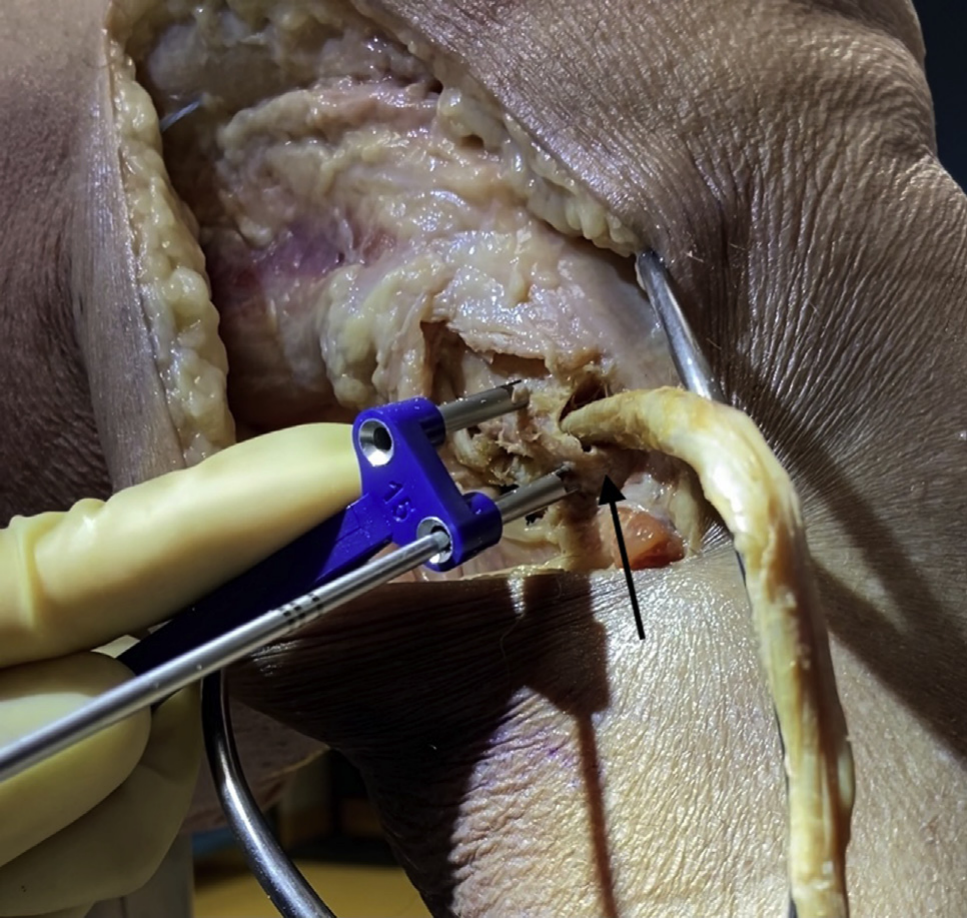
Intraoperative view of a right knee undergoing drill hole placement above and below (arrow) a lateralized fibular tunnel for posterolateral corner reconstruction prior to staple reinforcement. Guidewires are placed using a 15-mm DynaNite staple drill guide (Arthrex).

**Fig 6:**
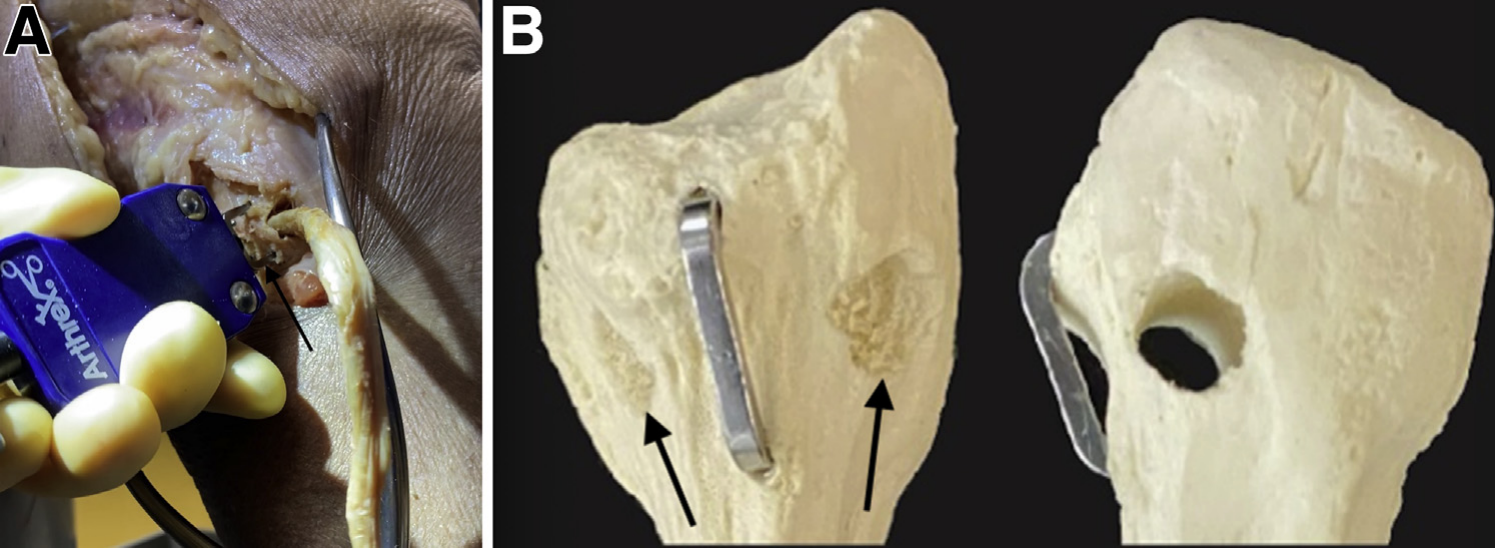
(A) Intraoperative view of a right knee undergoing implementation of a Nitinol staple for salvage of a lateralized fibular tunnel for posterolateral corner reconstruction. The 15-mm staple (arrow) is placed in holes drilled above and below the lateralized tunnel, compressed, and tamped down until it is flush with the cortical surface. (B) anterior-posterior (right) and lateral (left) views of a sawbone fibula, demonstrating a lateralized fibular tunnel for posterolateral reconstruction (arrows) that has been reinforced with a Nitinol staple.

**Fig 7:**
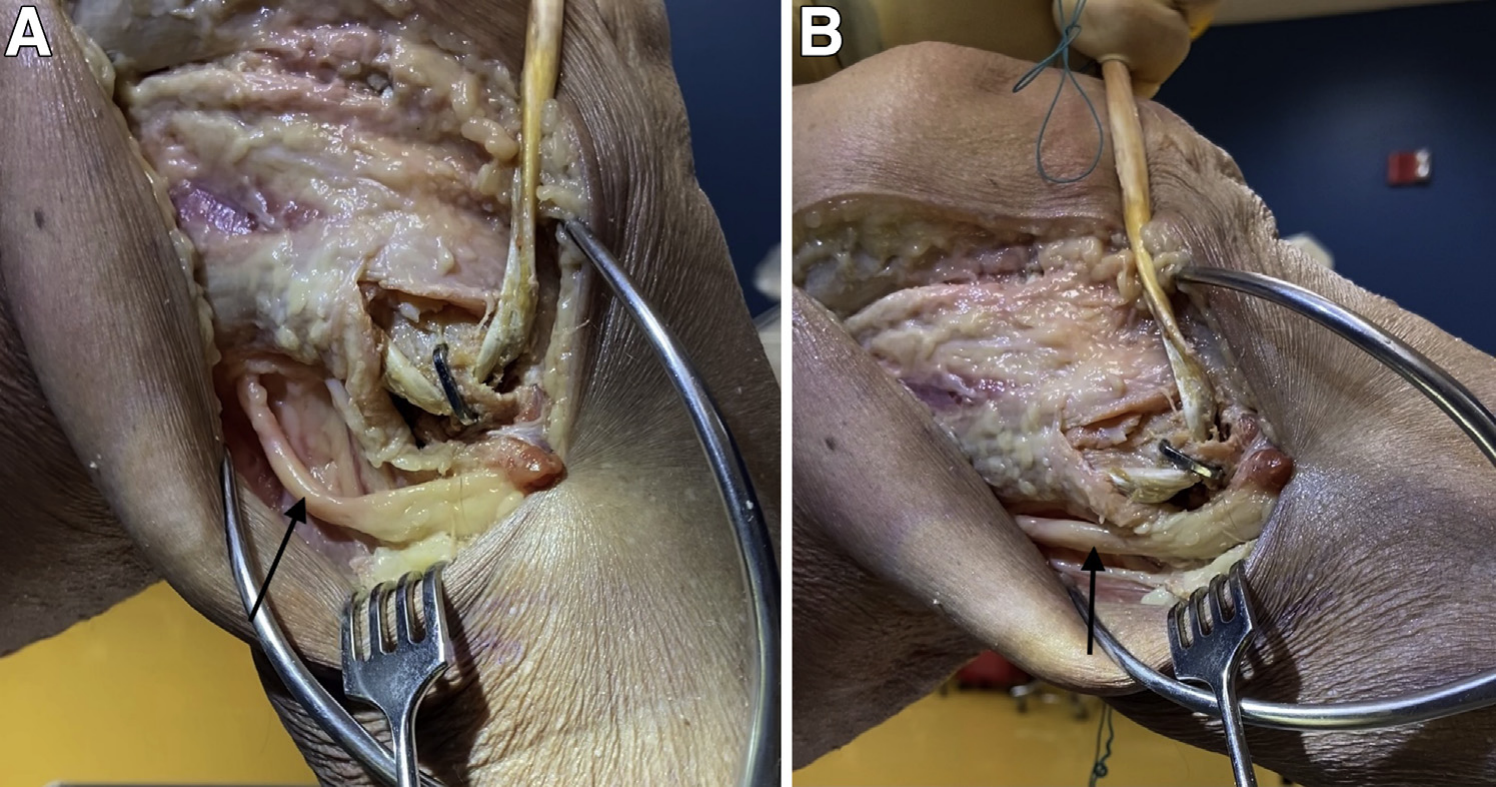
(A) A lateral intraoperative view of a right knee in 90° of flexion demonstrating the location of a Nitinol staple in the fibular head following reinforcement of a lateralized fibular tunnel for posterolateral corner reconstruction, with careful attention paid to its location in respect to the peroneal nerve (arrow). (B) A lateral intraoperative view of the right knee in full extension demonstrating the location of a Nitinol staple in the fibular head following reinforcement of a lateralized fibular tunnel for posterolateral corner reconstruction, with careful attention paid to its location in respect to the peroneal nerve (arrow).

After obtaining the appropriate materials (Fig 4), drill holes are placed above and below the lateralized fibular tunnel using a 15-mm staple drill guide (DynaNite Staple Drill Guide, Arthrex), and alignment pins can be used to identify the drill hole locations (Fig 5). The knob on the staple delivery device is turned so that the staple legs are open to a width equal to the predrilled holes. The alignment pins are then removed, and a single 15 × 15-mm compressive Nitinol staple (DynaNite Nitinol staple, Arthrex) is inserted in the lateral fibular neck and head with the delivery device, with the tines of the staple surrounding the top and bottom of the lateralized tunnel (Fig 6). Once the staple is inserted and seated against the bone, the delivery device knob can then be turned so that the staple is no longer under tension with the delivery device, effecting compression across each end of the fibular tunnel. The staple can then be fully seated in place using a staple tamp (DynaNite Staple Tamp, Arthrex). The augmented lateral fibular cortex is tested by passing the graft through the fibular tunnel and ranging the knee through flexion and extension while tension is held on the graft, ensuring no lateral cortical breach (Video 1, Fig 7). The low-profile nature of the staple ensures that there is minimal irritation to the peroneal nerve. The remainder of the posterolateral corner reconstruction may then be completed.^8^ The advantages and disadvantages of this technique are listed in Table 1; pearls and pitfalls are listed in Table 2.

**Table 1:** Advantages and Disadvantages

**Advantages**
Augmentation with nitinol staple provides compression across the tunnel, reducing risk of fibular head avulsion fracture and is mechanically strong.
Implementation of the staple is relatively simple and straightforward, with minimal steps and instrumentation needed to successfully perform this salvage technique.
The low-profile nature of the staple allows it to sit flush against the bone and limits the risk of irritation to surrounding soft tissue structures, including the peroneal nerve.
**Disadvantages**
Long-term outcomes for this salvage technique are unknown.
Hardware irritation may still occur.

**Table 2:** Pearls and Pitfalls

**Pearls**
Insertion of the staple should take place with the knee in flexion to minimize risk of injury to the peroneal nerve.
The staple should be sized so that the tines are placed on the superior and inferior poles of the fibular tunnel.
The knee should be ranged following staple implementation to ensure that the peroneal nerve does not contact the staple at full knee extension.
**Pitfalls**
Damage to the posterolateral corner graft can occur over time if staple tines erode through fibular tunnel wall and come in direct contact with the graft.
Leaving the staple proud over the lateral fibular cortex can lead to irritation of the peroneal nerve.
Additional drill holes that result from improper use of the drill guide can increase the risk of stress risers and subsequent fibular head fracture.

## Discussion

The success of PLC reconstruction is dependent on the accuracy of the anterolateral to posteromedial trajectory of the fibular head tunnel to obtain a mechanically robust and well-bordered tunnel. Because of the relatively small diameter of the fibular head, proper drilling of the fibular tunnel can be technically challenging, particularly if adequate posterolateral exposure around the fibular head is not performed. Familiarity with this procedure is highly variable within the surgical community, with less than 14% of orthopaedic sports medicine surgeons managing more than 10 PLC injuries per year.^9^ Technical difficulty and lack of familiarity with the procedure can both contribute to the high complication rate in PLC reconstruction; a recent systematic review demonstrated a complication rate of 20% in patients undergoing PLC reconstruction, with fibular head fracture being the most common intraoperative complication reported.^6^ However, salvage methods to treat the complications of PLC reconstruction have rarely been described.^8^

The use of a low-profile Nitinol staple for fracture fixation, osteotomy fixation, and arthrodesis in the hand, foot, and ankle has increased in recent years.^10^ The pseudo-elastic properties of the staple allow for sustained compression and have been shown to be superior to traditional fixation methods in fractures of the scaphoid and midfoot.^7^^,^^11^ Additionally, the low-profile nature of the staple minimizes contact and subsequent irritation to the peroneal nerve, even with full knee extension. Compared to other salvage techniques for a lateralized fibular head tunnel, including use of suture anchors and redrilling, the described method using a compression staple is mechanically superior and results in a higher ultimate tensile strength compared to controls in response to uniaxial tension testing (unpublished data).

### Conclusion

The low-profile nature, pseudoelastic compressive properties, and ease of insertion of the Nitinol staple make it a potential tool in the surgeon’s armamentarium for salvage of a lateralized fibular tunnel in posterolateral corner reconstruction of the knee. Clinical studies are warranted to validate the clinical efficacy of this technique.

## Supplementary Data

ICMJE author disclosure forms

Video 1 Following passage of the graft for posterolateral corner reconstruction, the graft is tensioned while the leg is cycled through flexion and extension. Upon recognition of a thinned lateralized fibular tunnel, the decision to reinforce the tunnel with Nitinol staple is made. Drill holes are placed above and below the fibular tunnel using a 15-mm staple drill guide. Alignment pins are removed, and a single 15 × 15 mm compressive Nitinol staple is placed in the lateral fibular neck and head, with the tines of the staple surrounding the top and bottom of the lateralized tunnel. Compression is achieved, and the staple can be further seated using a staple tamp. The graft is placed through the fibular head tunnel. The knee is then cycled through flexion and extension while tension is held on the graft to confirm mechanical stability of the construct.
